# Evaluation and selection of tandem repeat loci for a *Brucella *MLVA typing assay

**DOI:** 10.1186/1471-2180-6-9

**Published:** 2006-02-09

**Authors:** Philippe Le Flèche, Isabelle Jacques, Maggy Grayon, Sascha Al Dahouk, Patrick Bouchon, France Denoeud, Karsten Nöckler, Heinrich Neubauer, Laurence A Guilloteau, Gilles Vergnaud

**Affiliations:** 1 Centre d'Etudes du Bouchet BP3, 91710 Vert le Petit, France; 2 GPMS, Bât. 400, Institut de Génétique et Microbiologie, Université Paris Sud, 91405 Orsay cedex, France; 3 UR918 – Laboratoire de Pathologie Infectieuse et Immunologie, Institut National de la Recherche Agronomique, 37380 Nouzilly, France; 4 Institut Universitaire de Technologie, 29 rue du Pont Volant, 37082 Tours Cedex 2, France; 5 Bundeswehr Institute of Microbiology, Dept. of Bacteriology, Neuherbergstr. 11, D-80937 Munich, Germany; 6 Federal Institute for Risk Assessment, BfR, Diedersdorfer Weg 1, D-12277 Berlin, Germany

## Abstract

**Background:**

The classification of *Brucella *into species and biovars relies on phenotypic characteristics and sometimes raises difficulties in the interpretation of the results due to an absence of standardization of the typing reagents. In addition, the resolution of this biotyping is moderate and requires the manipulation of the living agent. More efficient DNA-based methods are needed, and this work explores the suitability of multiple locus variable number tandem repeats analysis (MLVA) for both typing and species identification.

**Results:**

Eighty tandem repeat loci predicted to be polymorphic by genome sequence analysis of three available *Brucella *genome sequences were tested for polymorphism by genotyping 21 *Brucella *strains (18 reference strains representing the six 'classical' species and all biovars as well as 3 marine mammal strains currently recognized as members of two new species). The MLVA data efficiently cluster the strains as expected according to their species and biovar. For practical use, a subset of 15 loci preserving this clustering was selected and applied to the typing of 236 isolates. Using this MLVA-15 assay, the clusters generated correspond to the classical biotyping scheme of *Brucella *spp. The 15 markers have been divided into two groups, one comprising 8 user-friendly minisatellite markers with a good species identification capability (panel 1) and another complementary group of 7 microsatellite markers with higher discriminatory power (panel 2).

**Conclusion:**

The MLVA-15 assay can be applied to large collections of *Brucella *strains with automated or manual procedures, and can be proposed as a complement, or even a substitute, of classical biotyping methods. This is facilitated by the fact that MLVA is based on non-infectious material (DNA) whereas the biotyping procedure itself requires the manipulation of the living agent. The data produced can be queried on a dedicated MLVA web service site.

## Background

Brucellosis is a zoonosis affecting animals and humans worldwide. *Brucella *infections may result in significant economic losses due to abortion and slaughtering of infected animals. Humans are mainly infected through the consumption of contaminated dairy products or by direct contact with infected animals. In addition, certain *Brucella *spp have to be considered as potential biowarfare agents. Six species are currently recognized, *B. abortus *(8 biovars), *B. melitensis *(3 biovars), *B. suis *(5 biovars), *B. ovis*, *B. canis *and *B. neotomae *[1]. More recently, *Brucella *strains have been isolated from marine mammals [2], suggesting the existence of additional species [3,4].

The genus *Brucella *is highly homogeneous (more than 90% DNA/DNA homology [5]). *Brucella *classification is mainly based on differences in pathogenicity, host preferences, and conventional microbiological tests used for phenotyping (biotyping) [6]. Routine identification of *Brucella *species and biovars still relies on biotyping (reviewed in [7]). Only a few tools exist for further molecular subtyping, of which none has proven to be fully satisfactory for epidemiologic investigations or tracing back strains to their origin. Tandem repeat (TR) sequences may be an interesting class of markers, since multiple alleles can be present at a single locus, and size differences are easily resolved by electrophoresis (reviewed by [8,9]). Tandem repeats are often classified as microsatellites (repeat units up to 8 bp) and minisatellites [10,11]. Tandem repeat typing has proven to be highly appropriate for the typing of pathogenic bacterial species with a high genetic homogeneity, including the *Mycobacterium tuberculosis *complex, *Bacillus anthracis*, and *Yersinia pestis *[12-15]. Recently, a family of tandem repeats located within a repeated sequence and present in multiple loci in the *Brucella *genome was used for strain typing [16,17]. The proposed set of eight microsatellite loci is extremely discriminant and highly efficient to distinguish strains within a local outbreak, but is unable to correctly predict the biovar or even the species of an isolate. A possible reason for that is the high mutation rate of these loci. Consequently, this MLVA assay cannot replace classical biotyping methods.

The availability of the whole genome sequences of *B. melitensis *16 M, *B. suis *1330 and *B. abortus *strain 9–941 [18-20] greatly facilitates the search for polymorphic DNA sequences [21]. In this report, we evaluated most tandem repeats showing at least two alleles among the three sequenced strains [22]. Eighteen reference strains and 3 strains isolated from marine mammals [23] were typed using these TR candidates to evaluate their associated polymorphism. For routine typing, a subset of 15 markers which enabled to cluster the isolates according to their biotype was selected. This set of markers was further evaluated on a collection of 236 isolates representing the major biovars affecting terrestrial mammals (Table 1) to produce a first reference data set [see Additional file 1] which can be queried via the internet [21,24].

## Results

### Evaluation of tandem repeats polymorphism

Comparison of the three genome sequences [21,22] identifies 107 TRs with a repeat unit larger than 5 bp and predicted to display size polymorphism. Eighty of them were evaluated for polymorphism among 21 reference and marine mammal strains (Table 1). Twenty-two TRs (numbered Bruce01 to Bruce22 in Table 2) have three predicted alleles. Twelve of the 22 are octamers, five of which have been previously characterized [16].

Typing was done by PCR using the set of primers listed in Table 2, as described [13]. Six markers failed to amplify DNA satisfactorily, and were not included in the further study: they generated multiple band profiles (bruce20-BRU329_8bp_148bp_7u; bruce38-BRU1116_18bp_108bp_2u; bruce71-BRU337_12bp_394bp_3u), or lacked amplification using the selected primers (bruce79-BRU163_12bp_141bp_4u), or no appropriate primers could be designed targeting the flanking regions because of the presence of repeated elements (bruce76-BRU243_21bp_2u; bruce77-BRU195_21bp_2u, not listed in Table 2).

**Figure 1:**
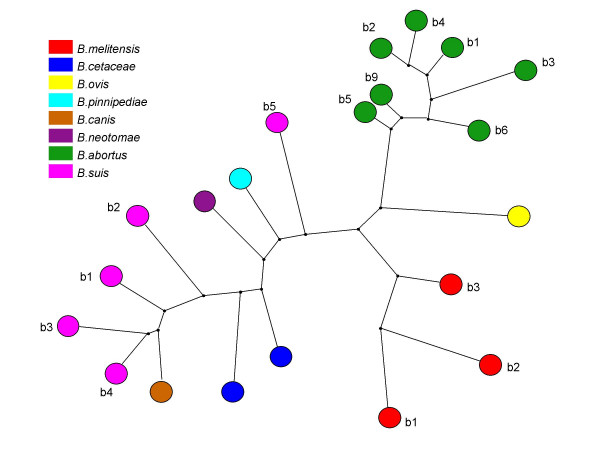
**Maximum parsimony analysis, on 21 reference strains using data from all 71 markers**. The different species are represented by different colours, as indicated. Biovars (b) are mentioned wherever relevant.

**Figure 2:**
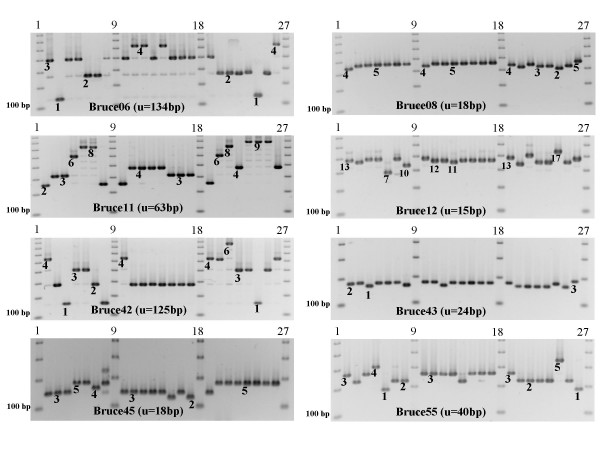
**Amplification patterns of MLVA panel 1 on the 21 reference strains**. Lanes 2- 8 = **2**: *B. melitensis*-bv1 (16M reference strain) ; **3**: *B. melitensis*-bv2; **4**: *B. melitensis*-bv3; **5**: *B. pinnipediae* B2/94; **6**: *B. cetaceae* B1/94; **7**: *B. cetaceae* B14/94; **8**: *B. ovis*. Lanes 10-17 = **10**: *B. melitensis*-bv1 (16M) ; **11**: *B. abortus*-bv1; **12**: *B. abortus*-bv2; **13**: *B. abortus*-bv3; **14**: *B. abortus*-bv4; **15**: *B. abortus*-bv5; **16**: *B. abortus*-bv6; **17**: *B. abortus*-bv9. Lanes 19-26 = **19**: *B. melitensis*-bv1 (16M); **20**: *B. suis*-bv1; **21**: *B. suis*-bv2; **22**: *B. suis*-bv3; **23**: *B. suis*-bv4; **24**: *B. suis*-bv5; **25**: *B. canis*; **26**: *B. neotomae*. Lanes 1;9;18;27 = **100bp DNA ladder.** The values for strain 16M are deduced from Table 2. The values for the other strains can be deduced from the 16M value, taking into account the indicated tandem repeat unit size. Examples are indicated, the full data can be deduced from the additional file [see Additional file 1].[Supplementary-material S1]

Three markers (bruce44-BRU256_12bp_110bp_3u; bruce65-BRU824_41bp_182bp_2u; bruce69-BRU488_57bp_181bp_1u) turned out to be monomorphic for the 21 reference strains. The results of the clustering analysis using the 71 remaining markers fits very well with the current knowledge of the degree of relationship between *Brucella *species [25] (Figure 1). We then looked for a subset of markers providing a similar discriminative power as the whole set for the collection of reference strains evaluated. Although extremely informative, the family of octamers, which includes the eight tandem repeats previously investigated [16,17], are not appropriate for species/biovar discrimination because of their hypervariability and more stable markers must be used. Among the other markers, a set of the ten most polymorphic loci clusters the different species as expected. Two of these ten markers display allele size ranges not appropriate for analysis on currently available automated DNA fragments sizing machines such as capillary electrophoresis sequencing machines (Bruce02 and Bruce15 have alleles up to 2 kb and 5 kb respectively). The amplification patterns of the 21 reference strains using the other eight TRs are shown in Figure 2. These 8 markers (Bruce06, 08, 11, 12, 42, 43, 45, 55) will subsequently be called MLVA typing panel 1. These are minisatellites loci with repeat units length above 9 bp [10]. In addition, 7 robust and highly polymorphic octamers (microsatellites) were selected to constitute MLVA typing panel 2. Panel 2 comprises Bruce04 (designated as TR6 in [16]), Bruce07, Bruce09 (TR8), Bruce16, Bruce18, Bruce21 and Bruce30 (TR2).

### Evaluation of a MLVA assay comprising 15 markers

**Figure 3:**
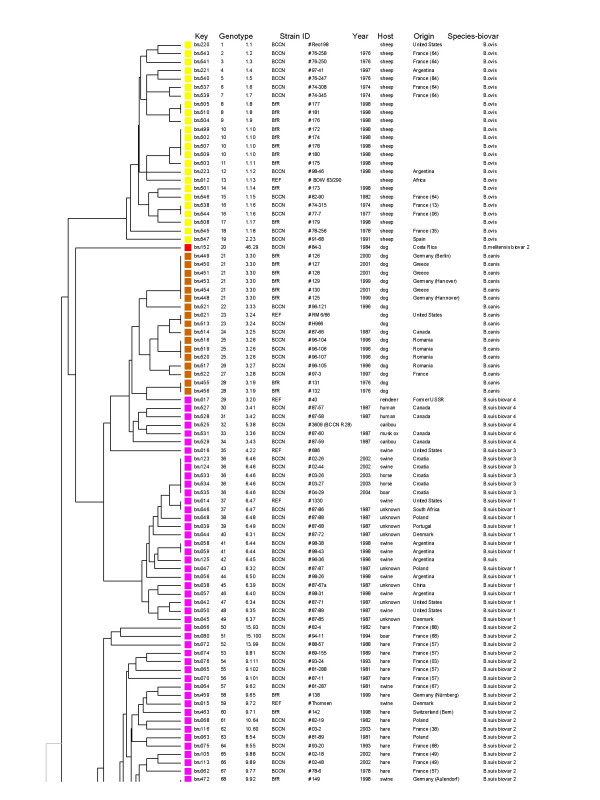
**Clustering analysis in 257 strains and isolates with the two panels of markers (MLVA-15), genotypes 1 to 68.** In the columns the following data are given from left to right : the DNA batch, the genotype, the strain ID including the name of the institution of origin (“BCCN” = *Brucella* Culture Collection from Nouzilly, INRA, Nouzilly, France ; “BfR” = Federal Institute for Risk Assessment, BfR, Berlin, Germany ; “REF” = the 21 reference and marine mammal strains, prepared by BCCN ; “vacc.” = vaccine strain ), year of isolation, host and geographic origin when known, species and biovar (when relevant). The first genotype number (going from 1 to 204) is the MLVA-15 genotype number. The second (for instance 1.1) indicates the panel 1 genotype number (from 1 to 51) followed by the panel 2 genotype number (from 1 to 200). The corresponding genotyping data can be found in the additional file [see Additional file 1]. Wherever possible, the more precise geographic origin within a country is indicated (for instance France (03) is a strain originating from the French department number 03 (Allier) in the centre of France). The first part of the clustering of the 257 isolates in 204 genotypes is presented. It comprises 68 genotypes, corresponding to *B. ovis*, *B. canis*, *B. suis* biovar 1, 3, 4, and part of the *B. suis* biovar 2 isolates. The colour code used is as shown in Figure 1.[Supplementary-material S1]

**Figure 4:**
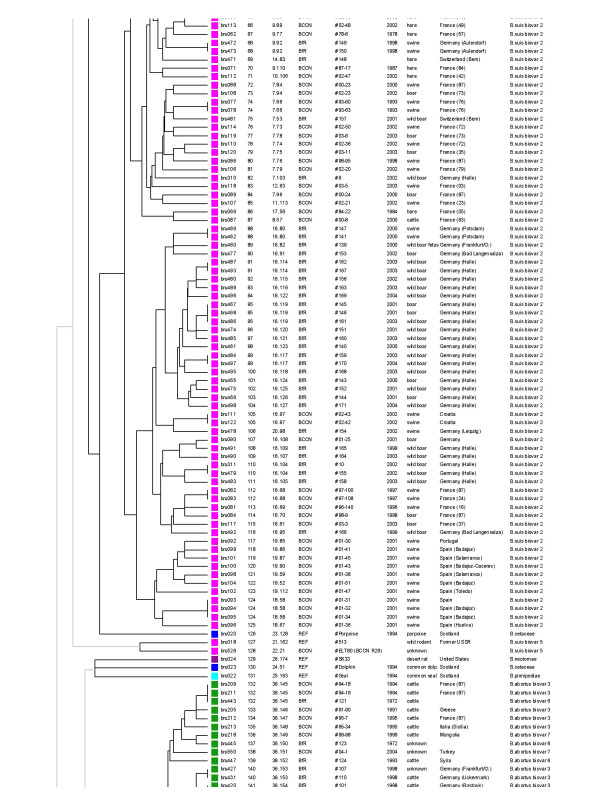
The columns content is as indicated in Figure 3 legend. The corresponding genotyping data can be found in the additional file [see Additional file 1]. The second part of the clustering is displayed, genotypes 66 to 141, comprising the rest of *B. suis* biovar 2 isolates, the *B. neotomae* strain, the three marine strains, the 2 *B. suis* biovar 5 isolates, and part of the *B. abortus* isolates. The colour code used is as shown in Figure 1

**Figure 5:**
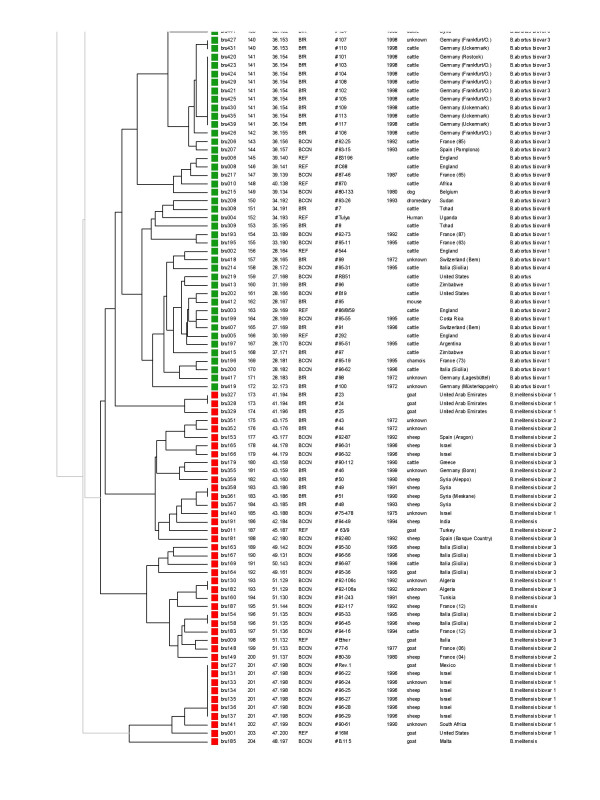
The columns content is as indicated in Figure 3 legend. The corresponding genotyping data can be found in the additional file [see Additional file 1]. The third part of the clustering (genotypes 140 to 204) is displayed, comprising the rest of *B. abortus* isolates, and the *B. melitensis* isolates. The RB51 vaccine strain tested here is genotype 159, S19 is genotype 161, and a number of Rev1 isolates share genotype 201. The colour code used is as shown in Figure 1.

The set of 15 TR markers (panel 1 and 2, listed with one or two asterisk in Table 2) was used for typing a larger collection of biotyped isolates including various species and biovars [see Additional file 1]. Among the 257 strains, panel 1 alone resolves 51 genotypes. This panel does not distinguish *B. suis *biovar 4 and *B. canis*. All *B. canis *strains investigated share panel 1 genotype 2 with some of the *B. suis *biovar 4 strains (Figure 3). Similarly, most *B. suis *biovar 3 strains share panel 1 genotype 4 with *B. suis *biovar 1. Panel 2 alone discriminates 200 genotypes. However, the resulting clustering only approximately fits with the expected species and biovar assignment. When using panel 1 and panel 2 together (MLVA-15 assay), 204 genotypes can be differentiated. The clustering analysis is shown in Figure 3, 4 and 5. A number of major clusters weakly connected to each other can be identified: *B. suis *biovar 1 (Figure 3), *B. suis *biovar 2( Figure 3 and figure 4), *B. abortus *(2 clusters, Figure 4 and Figure 5), *B. melitensis *(3 clusters, figure 5), *B. ovis* (Figure 3). *Brucella suis *biovar 5, *B. neotomae *and the marine mammal strains are quite distinct from the closest strains (Figure 4). *Brucella canis *and *B. suis *biovar 4 are closely related and loosely connected to the *B. suis *biovar 1 cluster (Figure 3). The three *B. melitensis *clusters fit moderately with the biotyping results. Similarly, *B. suis *biovar 3 strains do not constitute a consistent group.

## Discussion

The genus *Brucella *has been divided into species and biovars for a long time, but this classification has been discussed controversially since DNA-DNA hybridization has been applied. The genus proved to be highly monomorphic with a level of relatedness among all species higher than 90% [5]. This homogeneity complicated the development of molecular assays able to efficiently recognise the species-specific entities. This finding led to the proposal of a monospecies genus, i.e. *B. melitensis*. The classical species would be considered as biovars only. However, most bacteriologists did not accept this concept which has recently been rejected by the subcommittee of taxonomy [26]. The purpose of the present study was firstly to investigate the polymorphism of tandem repeat loci predicted to be polymorphic by comparing the data of the three different *Brucella *strains already sequenced and secondly to evaluate to which extend tandem repeat typing and classical biotyping clustering fit together. We evaluated most of these loci with a repeat unit of 5 bp or more.

Polymorphism has been confirmed at 71 loci. DNA was amplified at every locus from all 21 reference strains, including the 3 marine mammal strains (except for Bruce04 in the *B. melitensis *bv 3 reference strain Ether and Bruce01 in the *B.ovis* reference strain BOW63/290) confirming the very high genetic homogeneity of the genus *Brucella*.

A MLVA typing assay depends on the selection of markers which individually would not provide a relevant clustering. Taken separately, the TR markers are either not informative enough, or too variable or show a high level of homoplasy. However, the combination of well selected independent loci may be highly discriminatory and to some extend phylogenetically relevant, as shown previously for other species [9], and demonstrated here for *Brucella*. We propose a selection of 15 markers to be used in a *Brucella *MLVA assay consisting of two complementary panels, panel 1 (8 markers) and panel 2 (7 markers). The fifteen markers are a combination of moderately variable (minisatellites, panel 1) and highly discriminant (microsatellites, panel 2) loci (Table 2).

The strain clustering achieved is consistent with well-established phenotypic and molecular characteristics (Figure 3, 4 and 5). The biovars 1, 2 and 4 of *B. abortus *are gathered in agreement with (i) the sensitivity to thionin and (ii) the PCR-RFLP pattern of the *omp2a *genes specific for these biovars [27]. *B. abortus *biovar 3 strains are found in a separate group except for 2 strains originated from Africa (BCCN 93-26 and the reference strain Tulya). Strains isolated in Africa often show distinct phenotypes [28] and thus, it is not surprising to find these two strains separated. The two strains do not require CO_2 _for growing. Their MLVA closest neighbours are two *B. abortus *biovar 6 strains also isolated in Africa. Assignment to biovar 3 or 6 reflects the H_2_S production which is the unique phenotypical criteria to differentiate these two biovars. The MLVA assay confirms that some African strains significantly differ from isolates of other origin and that *B. abortus *biovar 3 is a heterogeneous group.

The *B. melitensis *group is very heterogeneous using either panel 1 or both panels (MLVA-15), and comprises four main subgroups. Biovar 2 and 3 strains are mixed in two groups, together with a few biovar 1 strains. The other biovar 1 isolates form 2 groups, one including the 16M reference strain, and the other (genotypes 173 and 174, Figure 5) comprising 3 isolates from the United Arab Emirates. *B. melitensis *BCCN 84-3 strain (MLVA-15 genotype 20) is an isolate from a dog in Costa Rica, which was biotyped as *B. melitensis *biovar 2, but appears to be distantly related to other *B. melitensis *strains. This strain is smooth as observed by the agglutination with anti-A serum, and the profile obtained in oxidative metabolism is typical of *B. melitensis*. Panel 1 analysis (not shown) does associate this strain with *B. melitensis*, but the full MLVA-15 analysis suggests a position closer to the *B. canis *group (Figure 3).

*B. suis *strains are clearly differentiated in three groups (Figures 3 and 4). A first group includes all biovar 1, 3, and 4 strains, and a second group all biovar 2 strains. The two rare biovar 5 strains are very distantly related. The correlation with biovars is good with some interesting exceptions. The five *B. suis *biovar 3 isolates from Croatia have the same genotype (MLVA-15 genotype 36, Figure 3 [see Additional file 1]), and cluster with *B. suis *biovar 1 strains but not with the reference *B. suis *biovar 3 strain. More *B. suis *strains phenotypically identified as biovar 3 from other geographic origins are required. This may suggest that the biovar 3 phenotype may have appeared independently more than once. Biovar 1 and biovar 3 strains are distinguished by sensitivity to fuchsine and ability to produce H_2_S. Atypical fuchsine-resistant biovar 1 strains have already been described [6], as well as atypical fuchsine-sensitive *B. melitensis *strains [29,30]. So both the fuchsine sensitivity, and the H_2_S production (as suggested above for *B. abortus*) may appear to be phylogenetically weak markers with some degree of homoplasy. Among biovar 2, strains isolated from Spain and Portugal are related and can be distinguished from other European strains investigated. Biovar 4 strains can be found right beside *B. canis*. Meyer [31] has previously proposed a model for evolutionary derivation of *Brucella *organisms on the basis of phenotypic characteristics and proposed a close relationship between *B. suis *biovar 3/4, and *B. canis*. PCR-RFLP analyses of the porin genes are in agreement with this finding [27].

Three classical vaccine strains were included, Rev.1 (genotype 201), S19 (genotype 161) and RB51 (genotype 159). Six other isolates, from Israel, share genotype 201. These streptomycin resistant isolates were confirmed as Rev.1 vaccine strains using the previously described assay [32] (data not shown). This is not unexpected since vaccination is used in this country, and simply illustrates the stability of the MLVA assay in the present case.

Strains clustering together frequently have a close or identical geographic origin, e.g. MLVA-15 genotype 16 comprises 2 *B. ovis *isolates, coming from the same region of France "Provence-Côte d'Azur" (departments 06 and 13). In almost all such instances where the MLVA genotype of two isolates is identical, the available epidemiological data is indeed compatible with a common source of infection. The rare exceptions would then suggest that some strains travel efficiently. MLVA-15 genotype 132 was observed in Germany in 1972 and in the centre of France (department 87) in 1994. MLVA-15 genotype 1 (*B. canis*) was observed in Greece and Germany. More epidemiological data will be needed in order to draw precise conclusions on the circulation of the strains.

The MLVA-15 results support the current classification of the genus *Brucella*. In addition, differences found by phenotypic identification and/or by molecular studies are also detected by MLVA. One major advantage of MLVA is the ease of data exchanges. The data itself can be summarized by a very simple flat text file containing the repeat copy numbers for each locus and each strain. This data can also be made accessible and queried across the internet as shown [21,24].

Another advantage is that MLVA typing only depends on the measurement of DNA amplicon sizes, so that a number of electrophoretic techniques can be used, ranging from manual, low-cost, agarose gels, to high-throughput capillary electrophoresis sequencing machines.

In the near future, it is tempting to speculate that international databases containing MLVA data of thousands of strains will be produced, and MLVA will become a routine assay for any new isolate. We believe that the MLVA-15 assay will be one step in this direction. A first use of the assay for a clinical application was recently described [33].

## Methods

### Bacterial strains

The 257 strains and isolates used for MLVA typing are listed or described globally in Table 1. One hundred and seventeen *B. suis*, 43 *B. melitensis*, 52 *B. abortus*, 24 *B. ovis*, one *B. neotomae*, 17 *B. canis *and 3 strains isolated from marine mammals [2] were investigated. This collection includes the 18 classical reference strains representing the different species and biovars of *Brucella*. All strains were mainly isolated from animals and in a few cases from humans or unknown species (Figure 3, 4 and 5), and were identified by phenotypical tests based on agglutination with monospecific antisera (serotyping), phage typing, dye sensitivity, CO_2 _requirement and H_2_S production [6].

### Identification of variable number tandem repeats by genomic sequence comparison

The methods previously described [10,12,21,22] and the genome sequence data for *B. suis *strain 1330, *B. melitensis *strain 16 M and *B. abortus *strain 9–941 [18-20] were used to identify TRs that may help to differentiate closely related genomes.

The different TRs are designated by using the nomenclature previously described [13]. For instance BRU211_63bp_257bp_2u (bruce11) is a TR at position 211 kb in the *B. melitensis *16 M genome. Its common laboratory name (alias name) is Bruce11. It has a 63 bp motif, and a total PCR product length of 257 bp in the *B. melitensis *16 M strain when using the primer set indicated in Table 2. This allele size corresponds to 2 units.

### PCR amplification and genotyping

*Brucella *DNA was prepared as previously described [27]. PCR amplification was performed in a total volume of 15 μl containing 1ng of DNA, 1× PCR Reaction Buffer, 1 U of *Taq *DNA polymerase (Qbiogen, Illkirch, France), 200 μM of each deoxynucleotide triphosphate, and 0.3 μM of each flanking primer as described previously [15].

Amplifications were performed in a MJ Research PTC200 thermocycler. An initial denaturation step at 96°C for 5 minutes was followed by 30 cycles of denaturation at 96°C for 30 s, primer annealing at 60°C for 30 s, and elongation at 70°C for 1 min. The final extension step was performed at 70°C for 5 min.

Two to five microliters of the amplification product were loaded on a 3% standard agarose gel for analyzing tandem repeats with a unit length shorter than 10 bp and on a 2% standard agarose gel for all others, and run under a voltage of 8 V/cm until the bromophenol blue dye had reached the 20 cm position. Gels were stained with ethidium bromide, visualized under UV light, and photographed (Vilber Lourmat, Marnes-la-Vallée, France). A 100-bp and a 20-bp ladder (EZ Load 100 pb or 20 bp PCR Molecular Ruler, Biorad, Marnes-la-Coquette, France) were used as molecular size markers depending on the tandem repeat unit length. Gel images were managed using the Bionumerics software package (version 4.0, Applied-Maths, Belgium).

### Data analysis

Band size estimates were converted to a number of units within a character dataset using Bionumerics version 4.0 (Applied-Maths, Belgium) [see Additional file 1]. Clustering analyses used the categorical coefficient and UPGMA (unweighted pair group method using arithmetic averages). The use of the categorical parameter implies that the character states are considered unordered. The same weight is given to a large or a small number of differences in the number of repeats at each locus. Maximum parsimony was done using Bionumerics, running 200 bootstrap simulations and treating the data as categorical.

### Polymorphism index

The Hunter Gaston diversity index [34] (HGDI) was used.

## Authors' contributions

MG, IJ, SAD, KN, HN were in charge of strain selection, collection and checking of related data, preparation and provision of DNAs. PLF did the MLVA genotyping work. GV was in charge of the Bionumerics database, error checking, clustering analyses. FD and PB did the genome sequence analyses for polymorphic tandem repeat searches and the genotyping page. GV wrote the report. IJ and MG helped to draft the manuscript. All authors read, commented and approved the final manuscript.

## Supplementary Material

Additional File 1 MLVA-15 data for each of the 204 genotypes. The first three columns from the left are genotype numbers obtained with the different panels. The subsequent columns are the typing data itself. The first 8 markers (headings, bruce06 to bruce55) constitute panel 1 (minisatellites, tandem repeat unit length above 9 bp). The last 7 columns (starting from bruce04) constitute panel 2 (microsatellites, tandem repeat unit length up to 8 base-pairs). Click here for file

## Figures and Tables

**Table 1:** *Brucella *strains studied (reference and field strains)

**Reference and marine strains**			
Species	Biovar	Strain	Host

*B. abortus*	1	544 (ATCC 23448; BCCN R4)^a^	Cattle
*B. abortus*	2	86/8/59 (ATCC 23449; BCCN R5)^a^	Cattle
*B. abortus*	3	Tulya (ATCC 23450; BCCN R6)^a^	Human
*B. abortus*	4	292 (ATCC 23451; BCCN R7)^a^	Cattle
*B. abortus*	5	B3196 (ATCC 23452; BCCN R8)^a^	Cattle
*B. abortus*	6	870 (ATCC 23453; BCCN R9)^a^	Cattle
*B. abortus*	9	C68 (ATCC 23455; BCCN R11)^a^	Cattle
*B. melitensis*	1	16 M (ATCC 23456; BCCN R1)^a^	Goat
*B. melitensis*	2	63/9 (ATCC 23457; BCCN R2)^a^	Goat
*B. melitensis*	3	Ether (ATCC 23458; BCCN R3)^a^	Goat
*B. suis*	1	1330 (ATCC 23444; BCCN R12)^a^	Swine
*B. suis*	2	Thomsen (ATCC 23445; BCCN R13)^a^	Swine
*B. suis*	3	686 (ATCC 23446; BCCN R14)^a^	Swine
*B. suis*	4	40 (ATCC 23447; BCCN R15)^a^	Reindeer
*B. suis*	5	513 (BCCN R21)^a^	Wild rodent
*B. ovis*		63/290 (ATCC 25840; BCCN R17)^a^	Sheep
*B. canis*		RM6/66 (ATCC 23365; BCCN R18)^a^	Dog
*B. neotomae*		5K33 (ATCC 23459; BCCN R16)^a^	Desert rat
*B. pinnipediae*		B2/94 (BCCN 94-73)	Common seal
*B. cetaceae*		B1/94 (BCCN 94-74)	Porpoise
*B. cetaceae*		B14/94 (BCCN 94-75)	Common Dolphin
**Overview of the 236 additional isolates**			

Species	Biovar	Number of isolates investigated	

*B. abortus*	1	14	
*B. abortus*	3	20	
*B. abortus*	4	1	
*B. abortus*	6	5	
*B. abortus*	7	2	
*B. abortus*	9	2	
*B. abortus*	(rough)	1	
*B. melitensis*	1	13	
*B. melitensis*	2	13	
*B. melitensis*	3	11	
*B. melitensis*	(atypical)	1	
*B. melitensis*	(rough)	2	
*B. suis*	1	13	
*B. suis*	2	87	
*B. suis*	3	5	
*B. suis*	4	5	
*B. suis*	5	1	
*B. suis*	(rough)	1	
*B. ovis*		23	
*B. canis*		16	
*total*		236	

^a^: Reference strain

ATCC, American type culture collection

BCCN, *Brucella *culture collection, Nouzilly, France

**Table 2:** List of tandem repeat loci investigated

**22 tandem repeats with a predicted different length in the 3 genomes:**											
**vntr**^a^	**alias name**^a^	**Chr**	***% ***^*b*^	upper primer	lower primer	**b.suis**^c^	**b.mel**^c^	**b.abor**^c^	**nb of different alleles**^d^	**min-max bp**^e^	***HGDI ***^*f*^

**BRU1938_8bp_371bp_9u**	**Bruce01 or TR7**	**1**	**100**	GGTCTGGGAAAACATGAAAAGC	AGCCGATCCTGCAAAACATAAT	**395**	**371**	**419**	**12**	**331–435**	**0.95**
**BRU1923_339bp_787bp_3u**	**Bruce02**	**1**	**94**	AACGCAGCATCACCAATGT	CCCAGATGTTCGGCTATAGTATG	**448**	**787**	**2143**	**6**	**448–1974**	**0.8**
**BRU1627_9bp_199bp_3u**	**Bruce03**	**1**	**82**	GGCTATTATTTCACCGGCAAGA	TCTTGATTTCCTTGCGATACCA	**208**	**199**	**217**	**4**	**154–217**	**0.48**
**BRU1543_8bp_152bp_2u**	Bruce04 or TR6**	**1**	**100**	CTGACGAAGGGAAGGCAATAAG	CGATCTGGAGATTATCGGGAAG	**184**	**152**	**160**	**8**	**152–208**	**0.87**
**BRU1365_8bp_185bp_3u**	**Bruce05**	**1**	**84**	AAGTATCAGGAAGGGCAGGTTC	GGGAGTAGGGGAATAAGGGAAT	**193**	**185**	**201**	**4**	**185–217**	**0.59**
**BRU1322_134bp_408bp_3u**	Bruce06*	**1**	**94**	ATGGGATGTGGTAGGGTAATCG	GCGTGACAATCGACTTTTTGTC	**274**	**408**	**542**	**4**	**140–542**	**0.73**
**BRU1250_8bp_158bp_5u**	Bruce07**	**1**	**100**	GCTGACGGGGAAGAACATCTAT	ACCCTTTTTCAGTCAAGGCAAA	**166**	**158**	**150**	**5**	**150–190**	**0.78**
**BRU1134_18bp_348bp_4u**	Bruce08*	**1**	**75**	ATTATTCGCAGGCTCGTGATTC	ACAGAAGGTTTTCCAGCTCGTC	**330**	**348**	**366**	**4**	**312–366**	**0.53**
**BRU588_8bp_156bp_7u**	Bruce09 or TR8**	**1**	**94**	GCGGATTCGTTCTTCAGTTATC	GGGAGTATGTTTTGGTTGTACATAG	**140**	**156**	**124**	**8**	**124–244**	**0.72**
**BRU221_19bp_127bp_2u**	**Bruce10**	**1**	**90**	ATCAATTCGCGGATATTTCACT	AGTGCGTTTCATATGTTTGCTG	**146**	**127**	**165**	**2**	**127–146**	**0.26**
**BRU211_63bp_257bp_2u**	Bruce11*	**1**	**80**	CTGTTGATCTGACCTTGCAACC	CCAGACAACAACCTACGTCCTG	**509**	**257**	**383**	**6**	**257–698**	**0.84**
**BRU73_15bp_392bp_13u**	Bruce12*	**2**	**58**	CGGTAAATCAATTGTCCCATGA	GCCCAAGTTCAACAGGAGTTTC	**345**	**392**	**375**	**7**	**302–452**	**0.82**
**BRU19_8bp_196bp_2u**	**Bruce13**	**2**	**100**	CGAACGATAGACCAGAACATGC	TTGAAAGAATCAGATAAGATAAAGCA	**204**	**196**	**220**	**10**	**196–300**	**0.85**
**BRU18_8bp_102bp_7u**	**Bruce14**	**2**	**100**	TTGCTTTATCTTATCTGATTCTTTCAA	GGTGTCGTTGGAGATAGAGGTC	**142**	**102**	**94**	**11**	**70–214**	**0.93**
**BRU1112_264bp_346bp_1u**	**Bruce15**	**2**	**81**	GCGGTGTTGTGTCTGTGGATA	GCCGTCAGTATCCACGTCATAG	**875**	**346**	**4112**	**7**	**346–2458**	**0.83**
**BRU548_8bp_152bp_3u**	Bruce16**	**2**	**100**	ACGGGAGTTTTTGTTGCTCAAT	GGCCATGTTTCCGTTGATTTAT	**168**	**152**	**176**	**8**	**144–240**	**0.85**
**BRU344_5bp_110bp_3u**	**Bruce17**	**2**	**100**	TTTTCACAGGGCATGTTCTCAG	CGCGTTTCGATTGTGGAAAATA	**125**	**110**	**115**	**6**	**110–135**	**0.79**
**BRU339_8bp_146bp_5u**	Bruce18**	**2**	**87**	TATGTTAGGGCAATAGGGCAGT	GATGGTTGAGAGCATTGTGAAG	**138**	**146**	**154**	**6**	**130–170**	**0.82**
**BRU324_6bp_163bp_18u**	**Bruce19**	**2**	**59**	GACGACCCGGACCATGTCT	ACTTCACCGTAACGTCGTGGAT	**169**	**163**	**184**	**7**	**76–190**	**0.79**
**BRU329_8bp_148bp_7u**	**Bruce20 or TR4**	**2**	**100**	AATACTGGGTCCAGTCCGATG	AGCGCAGCGACCATATTCT	**100**	**148**	**124**	**ND**	**ND**	**ND**
**BRU329_8bp_148bp_6u**	Bruce21**	**2**	**68**	CTCATGCGCAACCAAAACA	GATCTCGTGGTCGATAATCTCATT	**175**	**148**	**164**	**3**	**148–175**	**0.57**
**BRU322_8bp_158bp_6u**	**Bruce22 or TR1**	**2**	**98**	GATGAAGACGGCTATCGACTG	TAGGGGAGTATGTTTTGGTTGC	**150**	**158**	**134**	**10**	**125–214**	**0.9**

**56 tandem repeats with a predicted different length in 2/3 genomes:**											

**vntr**^a^	**alias name**^a^	**Chr**	***% ***^*b*^	upper primer	lower primer	**b.suis**^c^	**b.mel**^c^	**b.abor**^c^	**nb of different alleles**^e^	**min-max bp**^f^	***HGDI ***^*g*^

**BRU1990_9bp_152bp_1u**	**Bruce23**	**1**	**88**	ATCAGCGAGTCGAAGGTCAGTT	TTCGACTATGCCAATCCAGATG	**161**	**152**	**161**	**2**	**152–161**	**0.26**
**BRU1940_8bp_146bp_8u**	**Bruce24 or TR5**	**1**	**100**	AGGGGAGTATGTTTTTGGTTGC	GCTACAAGATCGAAGTGCTCCA	**146**	**146**	**106**	**10**	**106–217**	**0.88**
**BRU1915_8bp_215bp_2u**	**Bruce25 or TR3**	**1**	**100**	GGGAGTATGTTTTGGTTGCACA	CTATTTCGTCCTGCCATTCGAC	**239**	**215**	**239**	**8**	**215–736**	**0.9**
**BRU1704_12bp_189bp_5u**	**Bruce26**	**1**	**68**	TCTTCATCCTGCGAGATCATGT	ATTCGTGATCGGGGTGATGAT	**162**	**189**	**189**	**2**	**162–189**	**0.5**
**BRU1609_8bp_170bp_6u**	**Bruce27**	**1**	**76**	TCGACGTCGTCTGACATTTTCT	GGGAGTAAGGCAGTAGGGGAAT	**170**	**170**	**162**	**3**	**162–178**	**0.61**
**BRU1599_11bp_147bp_4u**	**Bruce28**	**1**	**95**	TATCTTCCACGGCCATGAATC	GGCAGGATCGGCGTATAGATAA	**136**	**147**	**136**	**2**	**136–147**	**0.1**
**BRU1528_15bp_132bp_3u**	**Bruce29**	**1**	**81**	TTGCGTTATTGATTGTCAGCAC	GCTGTGGCTCGTCTATGTGG	**132**	**132**	**117**	**2**	**117–132**	**0.47**
**BRU1505_8bp_151bp_6u**	Bruce30 or TR2**	**1**	**96**	TGACCGCAAAACCATATCCTTC	TATGTGCAGAGCTTCATGTTCG	**127**	**151**	**151**	**5**	**119–151**	**0.7**
**BRU1475_18bp_120bp_1u**	**Bruce31**	**1**	**100**	GCTGAATCTTTTCCGCATCCT	GGAATCTCTGCACTGACAAAGC	**138**	**120**	**120**	**2**	**120–138**	**0.51**
**BRU1424_8bp_106bp_5u**	**Bruce32**	**1**	**88**	AGGTTTCCGGCGATAATGG	TCGGGATGCGCTCTAGAATATC	**106**	**106**	**98**	**3**	**98–114**	**0.6**
**BRU1413_15bp_158bp_4u**	**Bruce33**	**1**	**81**	GATGGAGCTTGGTTCCTGCTT	GCATGATCCGTTTTCTTCTCAA	**143**	**158**	**143**	**3**	**128–158**	**0.5**
**BRU1409_18bp_83bp_2u**	**Bruce34**	**1**	**84**	GCGATCGAAGGAAATATCGAG	CGCTGCCGGGATGTGAAC	**101**	**83**	**101**	**2**	**83–101**	**0.26**
**BRU1282_10bp_136bp_4u**	**Bruce35**	**1**	**91**	TGCGATAACAGGTGTACCCAAG	GACGGCAGCCATGCTGAT	**116**	**136**	**116**	**3**	**116–136**	**0.27**
**BRU1234_15bp_157bp_4u**	**Bruce36**	**1**	**76**	TAAGGCTCTTGCGTTTGTATCG	TGCGTATCTTCAGACTGGCAAT	**142**	**157**	**142**	**2**	**142–157**	**0.38**
**BRU1176_21bp_124bp_2u**	**Bruce37**	**1**	**95**	CCAAGCGTATCATCGATCTGTC	TCGGACGCAGATTGTTTCTATC	**103**	**124**	**123**	**2**	**103–124**	**0.51**
**BRU1116_18bp_108bp_2u**	**Bruce38**	**1**	**100**	CTGAATTGGGAGGAGGAACCAG	AGCTGAACGACCTTGGCATCT	**126**	**108**	**108**	**2**	**108–126**	**ND**
**BRU1112_15bp_164bp_7u**	**Bruce39**	**1**	**66**	GAAGGTCTCGAAGGAAGAGCTG	CCATCCATATTGATCGTCAGGA	**164**	**164**	**146**	**2**	**146–164**	**0.51**
**BRU1048_15bp_94bp_2u**	**Bruce40**	**1**	**85**	AAAAGAAGGGTTTCCCCATACC	GGAAAGGACAGCTTCGAGTACC	**109**	**94**	**109**	**3**	**94–109**	**0.34**
**BRU1030_13bp_94bp_1u**	**Bruce41**	**1**	**100**	TTATGTCACCGCTGACGAATTT	CTCATTATGGACCCGGTCTTTC	**107**	**94**	**107**	**2**	**94–107**	**0.1**
**BRU424_125bp_539bp_4u**	Bruce42*	**1**	**96**	CATCGCCTCAACTATACCGTCA	ACCGCAAAATTTACGCATCG	**538**	**539**	**289**	**5**	**164–789**	**0.75**
**BRU379_12bp_182bp_2u**	Bruce43*	**1**	**69**	TCTCAAGCCCGATATGGAGAAT	TATTTTCCGCCTGCCCATAAAC	**170**	**182**	**182**	**3**	**170–194**	**0.55**
**BRU256_12bp_110bp_3u**	**Bruce44**	**1**	**96**	GGCGCAAGATCGGAATGC	AGGCAGGTGCTGATTCTCCT	**110**	**110**	**122**	**1**	**110**	**0**
**BRU233_18bp_151bp_3u**	Bruce45*	**1**	**70**	ATCCTTGCCTCTCCCTACCAG	CGGGTAAATATCAATGGCTTGG	**187**	**151**	**151**	**4**	**133–187**	**0.65**
**BRU217_15bp_256bp_4u**	**Bruce46**	**1**	**81**	AAAAGCTTCCGAACCAAGTGTC	GGAGCTGGTTGAGCGTTATTTC	**256**	**256**	**241**	**2**	**241–256**	**0.5**
**BRU149_15bp_116bp_2u**	**Bruce47**	**1**	**90**	CTGCCAAGGGCGAGATAAAC	CATCGTTCTGATCTTCGTGACC	**131**	**116**	**131**	**2**	**116–131**	**0.26**
**BRU131_29bp_131bp_2u**	**Bruce48**	**1**	**95**	TATAAGTCCAGCCCATGACAGG	GCGGAATATCTGGATGGGATAC	**131**	**131**	**160**	**2**	**131–160**	**0.51**
**BRU112_13bp_266bp_2u**	**Bruce49**	**1**	**97**	AACCTCGGTCTATGATGCAACC	ACGCAGGGTTAGGTTTCTCAAA	**279**	**266**	**265**	**2**	**266–279**	**0.52**
**BRU80_12bp_162bp_3u**	**Bruce50**	**1**	**75**	GCAGAACCTGATGAACAACCTG	ATTTTCTGGTCGAGATCGAAGG	**174**	**162**	**174**	**2**	**162–174**	**0.26**
**BRU80_15bp_74bp_2u**	**Bruce51**	**1**	**90**	TGACATGATGCAGAAAATGCAG	GTCCCTTGCCGCCTTTCAT	**74**	**74**	**89**	**2**	**74–89**	**0.47**
**BRU50_15bp_185bp_3u**	**Bruce52**	**1**	**95**	CAATGAACCAGATCAGCTTTCG	CGCCATGGTTTCAATATCACC	**170**	**185**	**170**	**2**	**170–185**	**0.26**
**BRU50_30bp_110bp_1u**	**Bruce53**	**1**	**70**	CGGTTATGGTGTGGAGCAACT	CTTCCAGCGGGCTTTCAG	**140**	**110**	**110**	**2**	**110–140**	**0.52**
**BRU28_13bp_150bp_1u**	**Bruce54**	**1**	**100**	CCGATCACAGACACAACAACTTC	GCGAAAAGGGAGCAGACATTAT	**163**	**150**	**163**	**2**	**150–163**	**0.26**
**BRU2066_40bp_273bp_3u**	Bruce55*	**1**	**80**	TCAGGCTGTTTCGTCATGTCTT	AATCTGGCGTTCGAGTTGTTCT	**234**	**273**	**273**	**5**	**194–354**	**0.69**
**BRU2028_12bp_135bp_4u**	**Bruce56**	**1**	**79**	TTGGTCGTTAGAACAAGAGTGG	CTGAACCTGTTCCGTCAAATCA	**135**	**135**	**123**	**2**	**123–135**	**0.38**
**BRU69_9bp_301bp_4u**	**Bruce57**	**2**	**90**	ATGGGAGCCTATTTCGCTTACA	GGCGGTAGAATGGATAGCTCAC	**292**	**301**	**292**	**3**	**283–301**	**0.19**
**BRU33_24bp_98bp_1u**	**Bruce58**	**2**	**82**	CATCCTGCTTGGTGTTCTTTTG	GATGGTCGTCACCAAGTCCAG	**122**	**98**	**122**	**2**	**98–122**	**0.32**
**BRU33_9bp_256bp_7u**	**Bruce59**	**2**	**78**	CGTATCATCCGGCAATGGT	CTTTCTCTTTGTCGTGGGCTTC	**265**	**256**	**256**	**2**	**256–265**	**0.38**
**BRU24_12bp_279bp_3u**	**Bruce60**	**2**	**90**	AGCAAATGAATATGTCGCGTTG	TTCACCCCGATATCGATGAAT	**267**	**279**	**279**	**2**	**267–279**	**0.38**
**BRU22_12bp_162bp_5u**	**Bruce61**	**2**	**75**	CCTAATTTCGCCATTCGGTAAC	TTGCGGATTTTCCGAATAGAAC	**162**	**162**	**150**	**2**	**150–162**	**0.5**
**BRU979_18bp_140bp_3u**	**Bruce62**	**2**	**85**	CTGACGCAGGGAAGCTTTGT	GAAAGAATGGTGAGCAGCAAGA	**155**	**140**	**155**	**2**	**140–155**	**0.26**
**BRU833_15bp_145bp_3u**	**Bruce63**	**2**	**77**	AGGGTGACATTTGTTGGAGTCA	GTGGACAGACCCATGGTAAACG	**160**	**145**	**145**	**2**	**145–160**	**0.51**
**BRU832_14bp_104bp_1u**	**Bruce64**	**2**	**93**	GAGACGACGCTTGAGGTTTTTC	CTTCCGGCGCTTCTTTCTTAT	**118**	**104**	**118**	**2**	**104–118**	**0.26**
**BRU824_41bp_182bp_2u**	**Bruce65**	**2**	**94**	CGCTCTAGGCATAGCTGTTGTG	GTCCAGCCGATAACCTTGCTAT	**143**	**182**	**143**	**1**	**142**	**0**
**BRU652_17bp_91bp_1u**	**Bruce66**	**2**	**100**	ATATCCATGCGGGAAGGAAGAT	TATGCAGTGGGCATTCTCTACG	**108**	**91**	**91**	**2**	**91–108**	**0.32**
**BRU609_31bp_155bp_2u**	**Bruce67**	**2**	**68**	GCACCAGTGCGAGAAAATAGTG	AATTTTCCTTCGCGACTCCTTT	**124**	**155**	**155**	**2**	**124–155**	**0.52**
**BRU564_18bp_111bp_3u**	**Bruce68**	**2**	**79**	GACAACGATCCGCAGAAAGG	GTCAATGCCCTGATCGGTATC	**111**	**111**	**93**	**2**	**93–111**	**0.47**
**BRU488_57bp_181bp_1u**	**Bruce69**	**2**	**100**	CGATGACAGAGCAAGACCGTTA	TTGACCGATAATTCTGCAATGG	**181**	**181**	**238**	**1**	**181**	**0**
**BRU339_21bp_146bp_2u**	**Bruce70**	**2**	**74**	GAGTAAGGCGAATAGGGGGAAC	AACTCTTCTTCCGCGACAACAC	**146**	**146**	**162**	**2**	**146–162**	**0.51**
**BRU337_12bp_394bp_3u**	**Bruce71**	**2**	**79**	GAAGACGGCTATCGACTGGTCT	ACAAGCTCTATTCGCCTTACGC	**370**	**394**	**370**	**ND**	**ND**	**ND**
**BRU322_8bp_230bp_8u**	**Bruce72**	**2**	**66**	GAAGACGGCTATCGACTGGTCT	GTTTCAATGAAGGCGAGGTGAG	**206**	**230**	**206**	**10**	**198–294**	**0.87**
**BRU285_28bp_178bp_3u**	**Bruce73**	**2**	**86**	GTGGAAGGCGTTGTCATTCTG	ATCGGTCATGGTCTATCCTTCC	**178**	**178**	**150**	**2**	**150–178**	**0.51**
**BRU275_8bp_147bp_6u**	**Bruce74**	**2**	**85**	GGATGAGGATTGAGGGCTTTT	ACGCAGACGATTTAACAAAGCA	**139**	**147**	**139**	**4**	**131–155**	**0.63**
**BRU250_19bp_82bp_2u**	**Bruce75**	**2**	**100**	AGGACTATCAGGTGCGTGACAA	AAGGAAGACGTCGCTGAAAGAC	**82**	**82**	**101**	**2**	**82–10**	**0.52**
**BRU181_14bp_122bp_2u**	**Bruce78**	**2**	**87**	CTAACAAATGACGGCAGAGTGC	TTGAACGCAAGCTTATCCAAAA	**136**	**122**	**136**	**2**	**122–136**	**0.32**
**BRU163_12bp_141bp_4u**	**Bruce79**	**2**	**65**	TCCTGTTGAACGCAAGCTAATG	ATACTTCAGGCGGGGAGGAC	**153**	**141**	**153**	**ND**	**ND**	**ND**
**BRU542_12bp_178bp_4u**	**Bruce80**	**2**	**87**	CGAGGAATGTCAGGAAGATCAC	ACACAGACGCCAAAAGACAAA	**166**	**178**	**166**	**2**	**166–178**	**0.18**

* markers constituting MLVA-15 ; the 8 non-octamers (*) are panel 1, the 7 octamers (**) are panel 2

^a ^naming nomenclature includes repeat unit size, PCR product size in strain 16M, corresponding repeat copy number, and common name;

^b ^internal repeat homogeneity ; ^c ^expected PCR product size in each of the three sequenced genomes, *Brucella suis *1330, *B. melitensis *16M, *B. abortus *9–941;

^d ^number of alleles observed in the 21 strains ; ^e ^observed size range ; ^f ^HGDI : Hunter-Gaston diversity index ; ND, not determined
